# Practices and factors associated with active management of the third stage of labor in East Africa: systematic review and meta-analysis

**DOI:** 10.1186/s12884-023-05761-9

**Published:** 2023-06-13

**Authors:** Kelemu Abebe Gelaw, Yibeltal Assefa, Belete Birhan, Natnael Atnafu Gebeyehu

**Affiliations:** 1 School of Midwifery, College of Health Science and Medicine, Wolaita Sodo University, Wolaita Sodo, Ethiopia; 2 School of Public Health, College of Health Science and Medicine, Wolaita Sodo University, Wolaita Sodo, Ethiopia; 3 Department of Psychiatry, College of Health Science and Medicine, Wolaita Sodo University, Wolaita Sodo, Ethiopia

**Keywords:** Practice, Factors, Third stage of labor, East Africa

## Abstract

**Background:**

Active management of the third stage of labor involves prophylactic uterotonics, early cord clamping, and controlled cord traction to deliver the placenta. It is designed to facilitate the delivery of the placenta by increasing uterine contractions during the third stage of labor. It is also used to prevent postpartum hemorrhage by averting uterine atony.This systematic review and meta-analysis’s emphasis was on the practice and factors associated with active management of the third stage of labor in East Africa.

**Methods:**

PubMed, Web of Science, Science Direct (Scopus), Google Scholar, African Journals Online, and the Cochrane Library electronic databases were used. Data were extracted using Microsoft Excel, and STATA version 14 was used for analysis. A p-value of 0.05 is regarded to indicate potential publication bias: the funnel plot, Begg, and Egger’s regression test were used to examine publication bias. Using I^2^ statistics, the heterogeneity of the studies was evaluated. Pooled analysis was carried out. By country, a subgroup analysis was conducted.

**Results:**

Thirteen studies were included in this systematic review and meta-analysis. The pooled prevalence of the practice of active management of the third stage of labor in East Africa was 34.42%. Received training (OR = 6.25, 95%CI = 3.69, 10.58), years of experience (OR = 3.66, 95%CI = 2.35, 5.71), and good knowledge (OR = 3.66, 95%CI = 2.35, 5.71) were statically associated with the practice of active management of third stage of labor.

**Conclusion:**

The pooled prevalence of practice for active management of the third stage of labor in East Africa was low. Factors that were statistically associated with the practice were received training, years of experience, and good knowledge. Obstetric care providers should continue to receive training in all components of active management of the third stage of labor through training and education programs.

**Supplementary Information:**

The online version contains supplementary material available at 10.1186/s12884-023-05761-9.

## Introduction

Labor is defined as a series of genital organ-related processes that expel fetuses from the uterus through the vagina into the outside world. There are four steps in the labor process. The first stage lasts until the cervix has fully dilated and begins with the initiation of true labor. The third stage of labor, which lasts between five and fifteen minutes and is distinguished by the delivery of the fetus and the expulsion of the placenta and membrane, is the most dangerous since there is a chance of heavy bleeding [1]. Although it lasts a short time, the third stage of labor can be particularly risky for mothers’ health [2].

Active management and expectant managements are the two types of management in the third stage of labor. After delivery of the fetus, Active Management Third Stage of Labor (AMTSL) is applied [3]. AMTSL is a useful intervention to lower the incidence of Post-Partum Hemorrhage (PPH) and has been pushed as part of initiatives to lower maternal mortality [4]. PPH is defined as the total blood loss ≥ 1000 ml within 24 h after the delivery process (including intrapartum loss) regardless of the route of delivery [5].

Three interrelated but separate components of active management of the third stage of labor include uterine massage, controlled cord traction, and prophylactic administration of uterotonic drugs [6]. Expectant management involves watching out for signs of placental separation and allowing the placenta to deliver spontaneously, or via the aid of gravity or nipple stimulation [7].

A randomized controlled trial was conducted to determine whether physiologic management or active management is more effective at preventing PPH. It showed that active management of the third stage of labor benefits mothers more than the physiological one [8]. Clinical trials showed that good practice of AMTSL was associated with about a 60–70% decrease in the incidence of PPH [9].

The most common birth-related complication is postpartum hemorrhage, which affects 2–4% [10] and 6% of vaginal and cesarean-section (C/S) deliveries [11]. PPH accounts for 25% of all maternal deaths worldwide; these deaths typically occur in the postpartum period, which is more common in low-income countries where there are fewer or unequipped birth attendants to practice AMTSL [12].

The risk for PPH is highest during the third stage of labor, because the uterus may not contract normally after giving the child. Uterine atony is the medical term for when the uterus does not resume normal contractions after giving birth. Uterine atony is the most common cause of PPH [13].

Based on the data collected from the study that has been studied, active management of the third stage of labor should become standard practice following straightforward vaginal deliveries in a health facility setting [14]. Active management of the third stage of labor can be implemented as a standard practice for a low cost, and it has a big clinical advantage in lowering maternal problems with little risk. Health facilities have uterotonic medications on hand for the management of postpartum bleeding [15].

The International Federation of Obstetrics and Gynecology’s (FIGO) recommendations are not followed by obstetric providers in low and middle-income countries performing AMTSL, especially when one considers that every birth attendant must possess an adequate level of knowledge and skills and access to adequate facilities [16]. Because the prevalence of PPH is increasing, it is unlikely that obstetric care providers will use AMTSL intervention. However, studies have found a gap in the AMTSL’s application [17].

Task shifting has been recommended as a way to improve the implementation of AMTSL, but there are several obstacles to overcome [18]. According to research done in seven Sub-Saharan African (SSA) countries, AMTSL was only correctly achieved in 0.5–32% of the recorded deliveries [19]. Several studies focusing on the prevalence of practices and factors associated with active management of the third stage of labor in East Africa have been published. However, there is no systematic review, and meta-analysis to summarize evidence prevalence of practices and factors associated with active management of the third stage of labor in East Africa.

Generally, this finding will provide more evidence that is relevant to the improvement and implementation of AMTSL in Eastern African countries. It will enable us to suggest factors and risks which alleviate the practice of AMTSL among obstetric care providers. It is also very important to inform health organizations and other concerned bodies to identify areas of poor practice of AMTSL and to provide decisive and reactive action when required. This systematic review aimed to assess practice and associated factors with active management of the third stage of labor in Eastern African countries.

## Methods

### Search strategy

An extensive data search was performed on PubMed, Web of Science, Scopus, Google Scholar, Cochrane Library, and African Journals Online (AJOL) databases used to get the research articles. Searching strategies were established by using Boolean operators (“OR” or “AND”) and the following key terms: practice, associated factors, labor third stage, and East Africa. The search strategies for Google Scholar were: “practice” “associated factors” and “active management third stage of labor”.

The search strategy made in PubMed was: (((((“Knowledge“[Text Word] OR “awareness“[Text Word] OR “Knowledge“[MeSH Terms]) AND “Practice“[Text Word]) OR “Practice“[Text Word] OR “determinants“[Text Word]) AND “OR“[All Fields] AND “active management“[Text Word]) OR “third stage of labor“[Text Word]) AND (“Africa south of the east“[MeSH Terms] OR (“Africa“[All Fields] AND “east“[All Fields]. We used PICO questions that had been modified to follow the &quot; PEO&quot;(Population, Exposure, and Outcome) style for the explicit presentation of our review question and the explicit clarification of the inclusion and exclusion criteria. The search period was from April 10/2023 to April 24/2023. We followed the Preferred Reporting Items for Systematic Reviews and Meta-Analyses (PRISMA) criteria for conducting the systematic review [20].

### PECO guide

#### Population

Obstetric care providers.

#### Exposure

Obstetric care providers who had practiced AMTSL.

#### Outcome

The practice of Active management of the third stage of labor.

### Eligibility criteria

Studies were included if they reported an observational study on the variables affecting the early resumption of sexual activity for postpartum women, described the techniques used to evaluate such activity, were available in full-text, were conducted on postpartum women, no years of publication were limited, and were published in English. Studies were excluded if unrelated research works; studies without sufficient data; duplicate sources; pieces of research with unclear methods; interventional studies; case reports; articles whose full text was not available: an attempt was made to contact the corresponding author; review articles. The COCOPOP(Condition, Context, and Population) paradigm was utilized to determine the suitability of the included studies for this investigation. Obstetric care providers who had practiced made up the study’s population (POP), while the prevalence of practice served as the condition and East Africa served as the setting.

#### Operational definitions

##### Active management of the third stage of labor (AMTSL)

is the use of oxytocin for delivery of the anterior shoulder; early clamping and cutting of the cord; nipple stimulation by commencing breastfeeding immediately after delivery; assisted delivery of the placenta through controlled cord traction and massaging of the uterus immediately after delivery.

##### Good practice

Obstetric care providers who administer oxytocin within 1 min, apply CCT, and perform uterine massage correctly in a proper sequence.

#### Study selection and data extraction

Retrieved articles were exported to the reference manager software; endnote software was used to remove duplicate studies. Three independent reviewers screened the title and abstract (KA, YA, and NA). The disagreement was handled based on one established article selection criteria. Data were extracted using a standardized data extraction format prepared in Microsoft Excel by two independent authors (KA and BB). Any difference during extraction was solved through discussion. Name of the first author, country, study design, year of publication, study setting, sample size, and prevalence of knowledge and practice on active management of the third stage of labor in East African countries were collected.

### Study population

Obstetric care providers.

### Quality assessment

The scientific validity and quality of each study were evaluated using Joanna Briggs Institute’s (JBI) quality assessment approach, designed for cross-sectional research quality assessment methods. Each author assessed each study separately using the assessment methods. Analyses were performed on assessment scores that (4 out of 8) satisfied a quality rating requirement of 50%. The average score of the investigators’ quality evaluation outcomes was taken into account to manage a score disparity between them (Supplementary 2). Two independent authors (KA and YA) appraised the quality of the study. Any disagreement raised during the risk of bias assessment was resolved through a discussion led by the third author (NAG) (Supplementary 3).

### Publication bias, heterogeneity, and statistical analysis

The data were extracted using Microsoft Excel and analyzed using STATA 14 statistical software. The presence of significant between-study heterogeneity was assessed using Cochrane Q and I^2^ statistics. The presence of heterogeneity was illustrated by a forest plot. We utilized a random-effects model for analysis to estimate the pooled effect because we found a high level of heterogeneity. Analysis of the subgroups was done by study setting, study design, and country. A sensitivity analysis was carried out to determine the effect of one study’s findings on the overall estimate. To detect the presence of considerable heterogeneity, meta-regression was computed based on the year of publication, country, and sampling type. Publication bias was checked by funnel plots and Egger’s regression tests. At a p-value of less than 0.05, publication bias was considered statistically significant. We used adjusted odds ratio estimates with confidence intervals (CI) as a measure of association. A random effect model was used to assess the overall effect of practice, which was then measured by the prevalence rates and odds ratio with a 95% CI. The result was presented in the form of text, tables, and figures.

## Result

### Selection of included studies

**Fig. 1:**
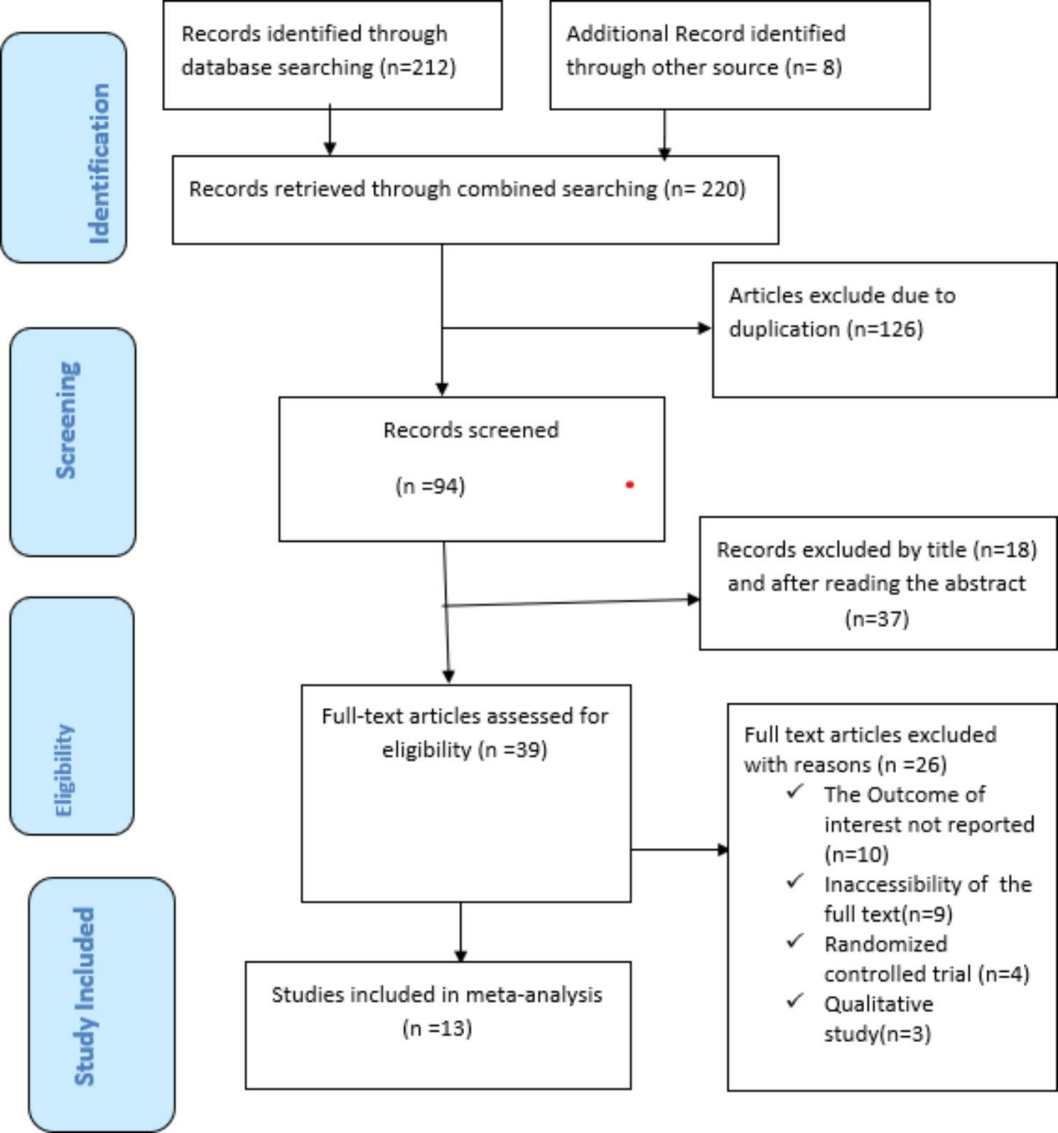
PRISMA flow diagram showing studies used for Systematic Review and Meta-analysis of practices and factors associated with active management of the third stage of labor in East Africa

Database search resulted in a total of 220 research articles. For these studies, 126 duplicate articles were removed, and 55 studies were excluded after reviewing their titles and abstracts. At the eligibility evaluation phase, out of the remaining 39 articles, 26 articles were removed after examining their full text, and similarly by considering the inclusion and exclusion criteria. Finally, 13 studies [21–33] with 2,158 participants were included in the analysis (Fig. 1).

### Characteristics of included studies

Table 1 displays the characteristics of all included studies: the first author’s name, publication year, study setting, study design, sample size, country, and the percentage of the practice of active management of the third stage of labor. In terms of country-wise distribution, the included 22 studies were comprised of 6 from Tanzania [34–39], 5 from in Ethiopia [22–26], and 2 from in Uganda [36, 37]. All selected studies were assessed using methodological quality checklists based on The Joanna Briggs Institute (JBI) systematic review checklist (Supplementary file 1). None of the studies were excluded based on the quality assessment criteria (Table 1).

**Table 1:** Description of the studies used in the systematic review and meta-analysis of knowledge, practice, and factors associated with active management of the third stage of labor in East Africa

First Author/Year	Study setting	Country	Study design	Good Practice (%)	Sample size	Sampling type	Study Quality
RahelY et.al/2015 [21]	Institution	Ethiopia	Cross-sectional	47.1	136	None probability	Low risk
Biresaw W eta.al 2021 [22]	Institution	Ethiopia	Cross-sectional	48.1	356	Probability	Low risk
Getu E et.al/2020 [23]	Institution	Ethiopia	Cross-sectional	43.5	278	Probability	Low risk
Wondwosen M et. al/2021 [24]	Institution	Ethiopia	Cross-sectional	32.5	254	Probability	Low risk
Aregahegn W et. al/2019 [25]	Institution	Ethiopia	Cross-sectional	29.8	171	Probability	Low risk
Godfrey S et.al/2009 [26]	Institution	Tanzania	Cross-sectional	7	106	Probability	Low risk
JohoAet .a/2019 [27]	Institution	Tanzania	Cross-sectional	38.4	172	Probability	Low risk
FatinaBet.al/2020 [28]	Institution	Tanzania	Cross-sectional	46.8	160	None probability	Low risk
MuyangaDet.al/2019 [29]	Institution	Tanzania	Cross-sectional	45	340	None probability	Low risk
FatinaRet.al/2011 [30]	Institution	Tanzania	Cross-sectional	10	105	Probability	Low risk
Haule M/2015 [31]	Institution	Tanzania	Cross-sectional	19.6	105	None probability	Low risk
Sangay B et.al/2018 [32]	Institution	Uganda	Cross-sectional	47.5	40	None probability	Low risk
Abalo J/2018 [33]	Institution	Uganda	Cross-sectional	35	40		Low risk

### Meta-analysis

#### Prevalence of practice of active management of the third stage of labor in East Africa

**Fig. 2:**
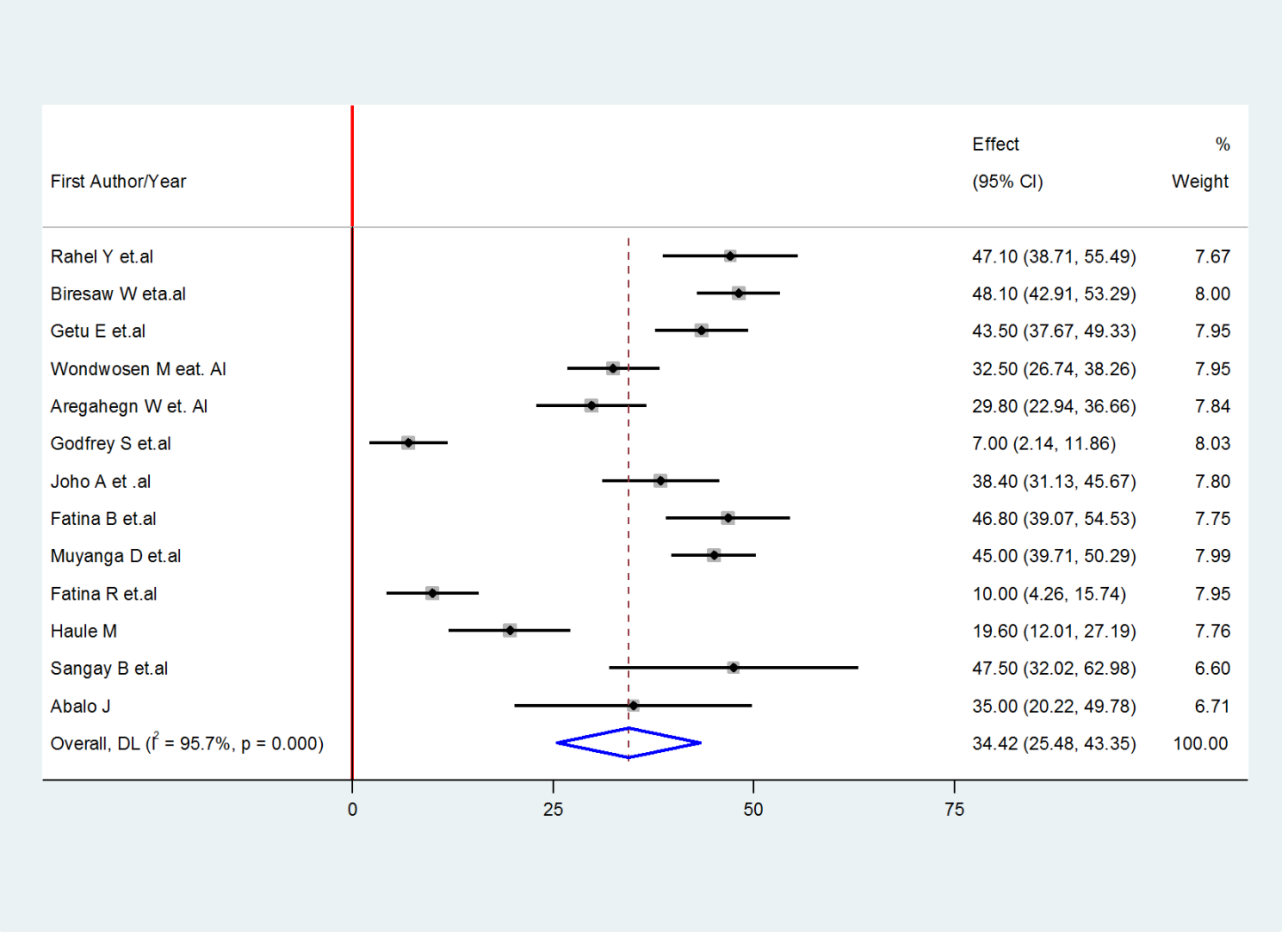
The Pooled prevalence of practice for active management of the third stage of labor in East Africa

Based on the reviewed studies, the prevalence of practice for active management of the third stage of labor ranged from 7 to 48.1% [22, 26]. The pooled prevalence of practice for active management of the third stage of labor was 34.42% (95% CI 25.48, 43.35). The random-effect model was used to analyze the pooled prevalence, however, a high and significant heterogeneity for the included studies (*I*^2^ = 95.7%; P-value ≤ 0.001) was observed (Fig. 2).

#### Subgroup analysis for practice

After confirming the presence of heterogeneity in the studies, subgroup analysis was done utilizing the study setting and study design to identify the source of heterogeneity. Nevertheless, there was still proof of study heterogeneity. Subgroup analysis based on country, the pooled prevalence of practice for active management of the third stage of labor in East Africa, the highest was in Uganda (41.031%), and based on sampling the highest was to none probability (40.89)(Table 2).

**Table 2:** The pooled prevalence of practice for active management of the third stage of labor in East Africa

Country	Random effects(95%CI)	Test of heterogeneity I²
Ethiopia	40.169 (32.719, 47.618)	85.9%
Tanzania	27.724(12.587, 42.860)	97.2%
Uganda	41.031(28.789,53.274)	23.7%
Overall	34.416 (25.481, 43.351)	100.00
**Sampling type**		
Probability	32.160(16.583,47.738)	97.4%
None probability	40.897 (29.922,51.872)	89.4%
Overall	34.378(25.032,43.724)	96.0%

#### Sensitivity analysis

**Fig. 3:**
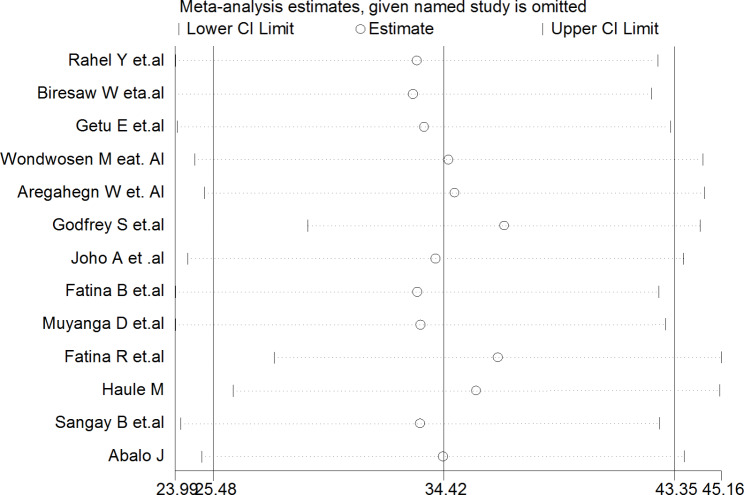
Sensitivity analysis of the practice for active management of the third stage of labor in East Africa

Sensitivity analysis was carried out to detect the effect of each study on the pooled prevalence of practice for active management of the third stage of labor in East Africa by excluding one study at a time. The results of the random effect model reported that omitted studies had shown a significant impact on the overall estimate of active management of the third stage of labor (Fig. 3).

#### Meta-regression

By publication year, country, and sampling type, Meta-regression was conducted to look into the cause of heterogeneity. There was no proof that the publication year (p = 0.492), country (p = 0.754), and sampling type (p = 0.177) were the sources of heterogeneity (Table 3).

**Table 3:** Meta-regression analysis based on year of publication, country, and sampling type

The possible source of heterogeneity	Coefficient	Standard error	P value
Publication year	1.163	0.247	0.492
Country	0.580	0.984	0.754
Sampling type	0.823	0.873	0.177

#### Publication bias

**Fig. 4:**
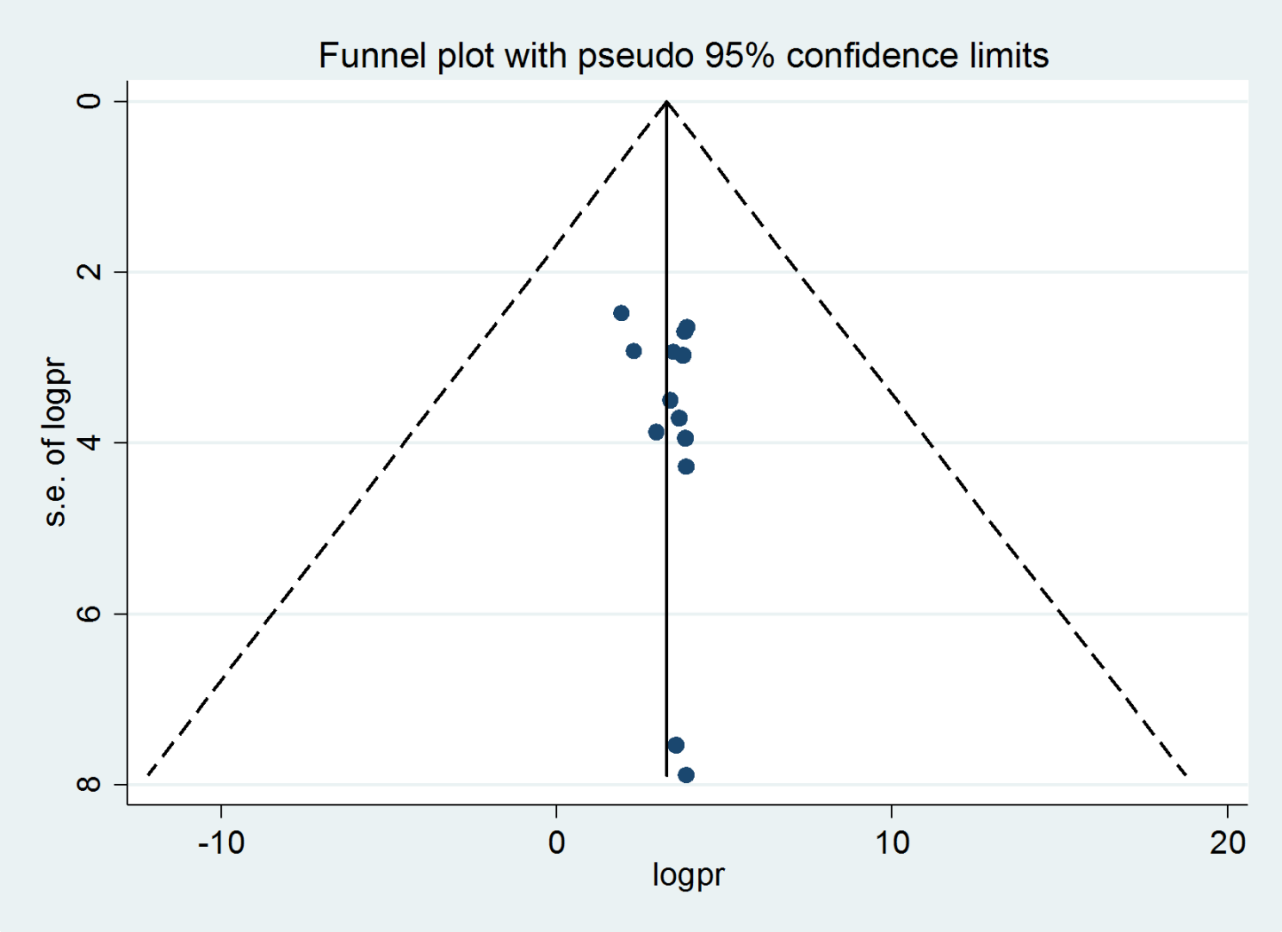
Funnel plots for publication bias of practice for active management of the third stage of labor in Sub-Saharan Countries

A funnel plot, an Eggers regression test, and a Begg’s test were used to determine whether there was publication bias across the studies. With a p-value of 0.360and 0.386, respectively, the results of Eggers regression test and Begg’s test revealed an absence of publication bias. The finding of the funnel plot also shows the absence of publication bias across the studies (Fig. 4).

#### Factors associated with the practice of active management of the third stage of labor

**Fig. 5:**
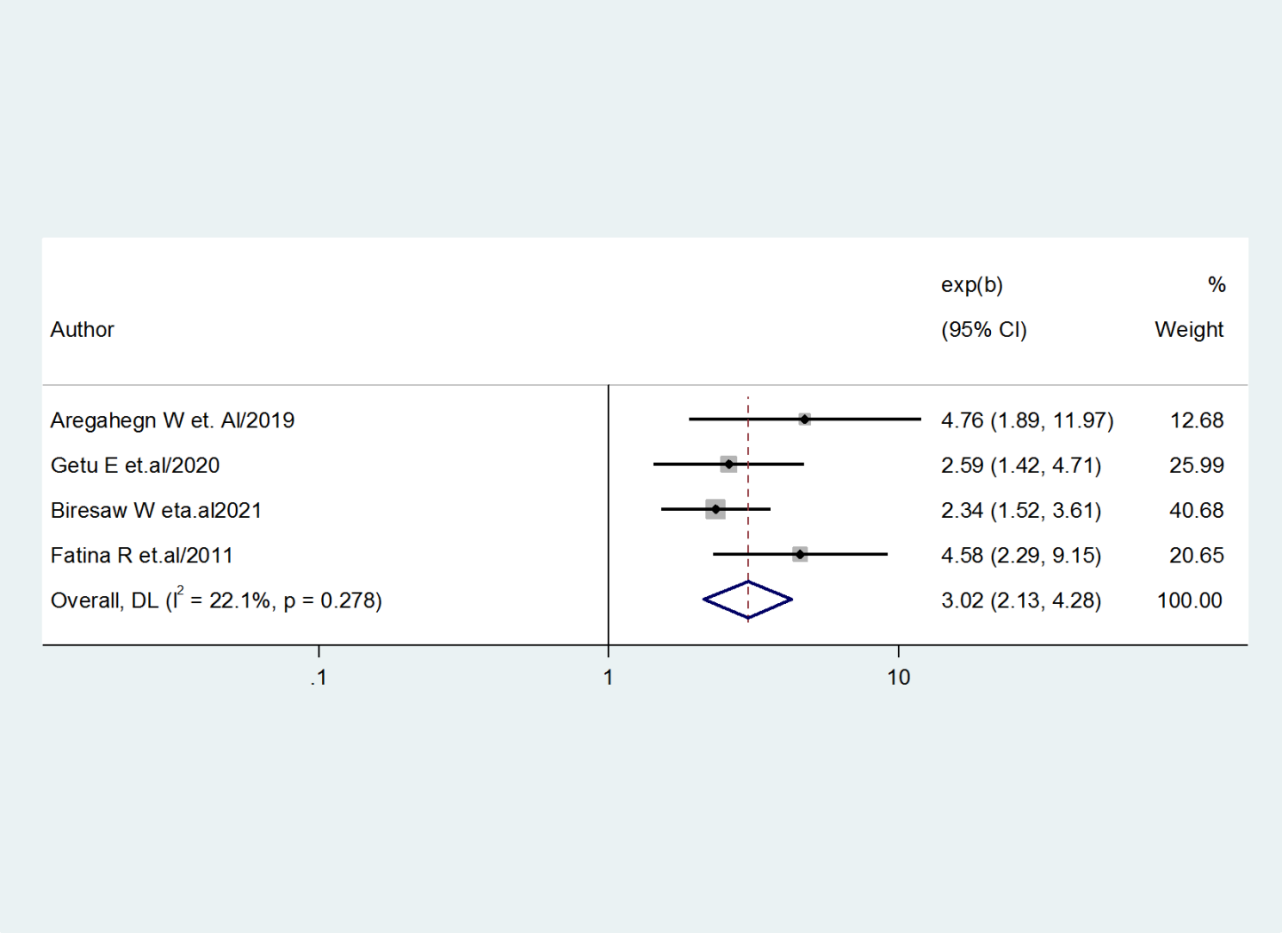
Forest plot showing the association between the practice of active management of the third stage of labor and received training

Four studies [22, 23, 25, and 30] were used to assess the association between the prevalence of good practice for AMTSL and receiving training. The results revealed that the pooled effect of received training was significantly associated with AMTSL. Obstetric care providers who had received in-service training were three times more likely to practice AMTSL than who did not receive (OR = 3.02, 95%CI = 2.13, 4.28). The study did not show any evidence of heterogeneity (I-squared = 22.1%, p = 0.278) (Fig. 5).

**Fig. 6:**
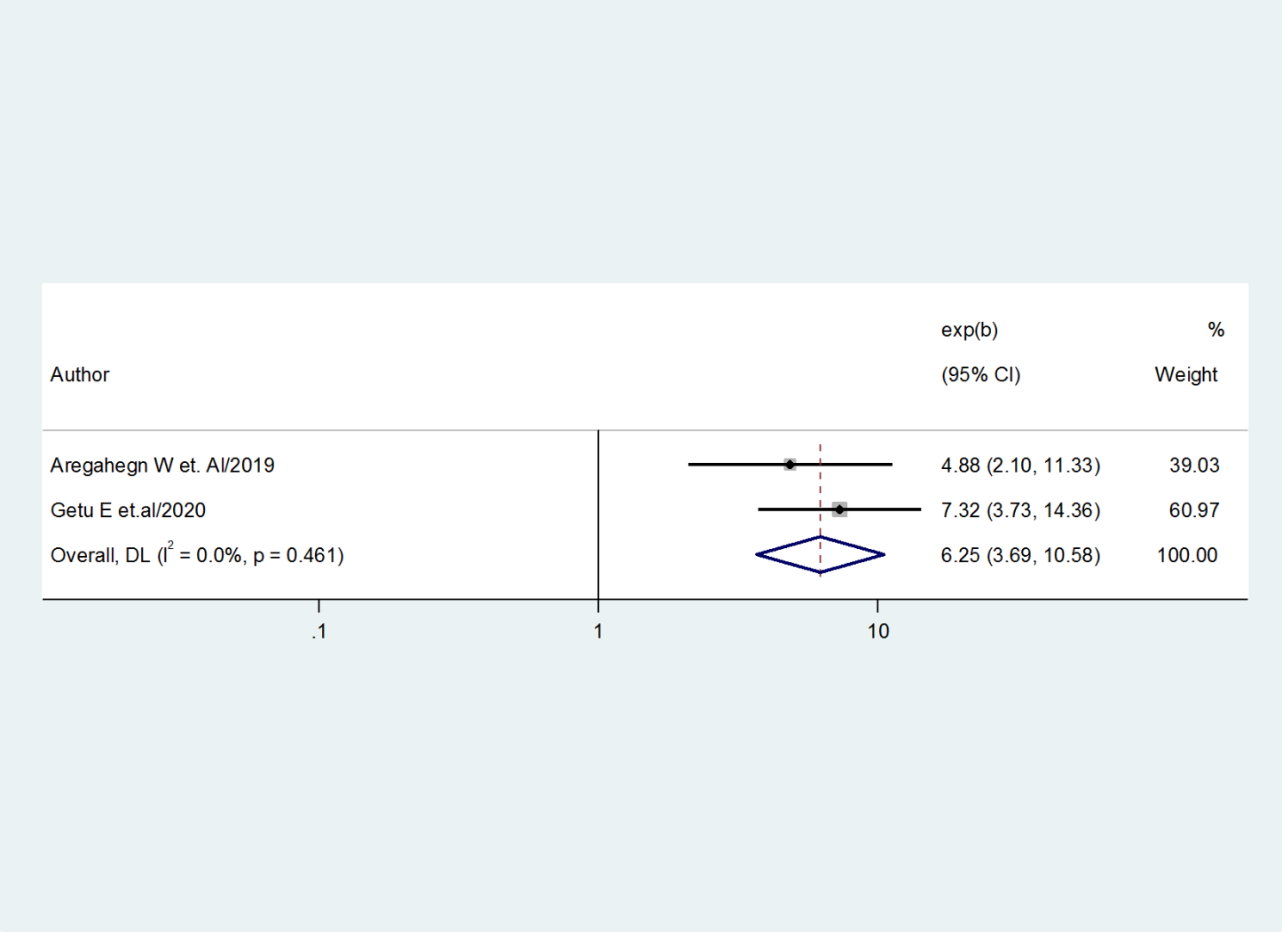
Forest plot showing the association between good practice for active management of the third stage of labor and good knowledge

Two studies [23] and [25] were used to analyze the association between the prevalence of good practice for AMTSL and good knowledge. The results revealed that the pooled effect of good knowledge was significantly associated with AMTSL. Obstetric care providers who had good knowledge were six times practice AMTSL than who had to poor knowledge (AOR = 6.25, 95%CI = 3.69, 10.58). The studies did not show any evidence of heterogeneity (I-squared = 0.0%, p = 0.461) (Fig. 6).

**Fig. 7:**
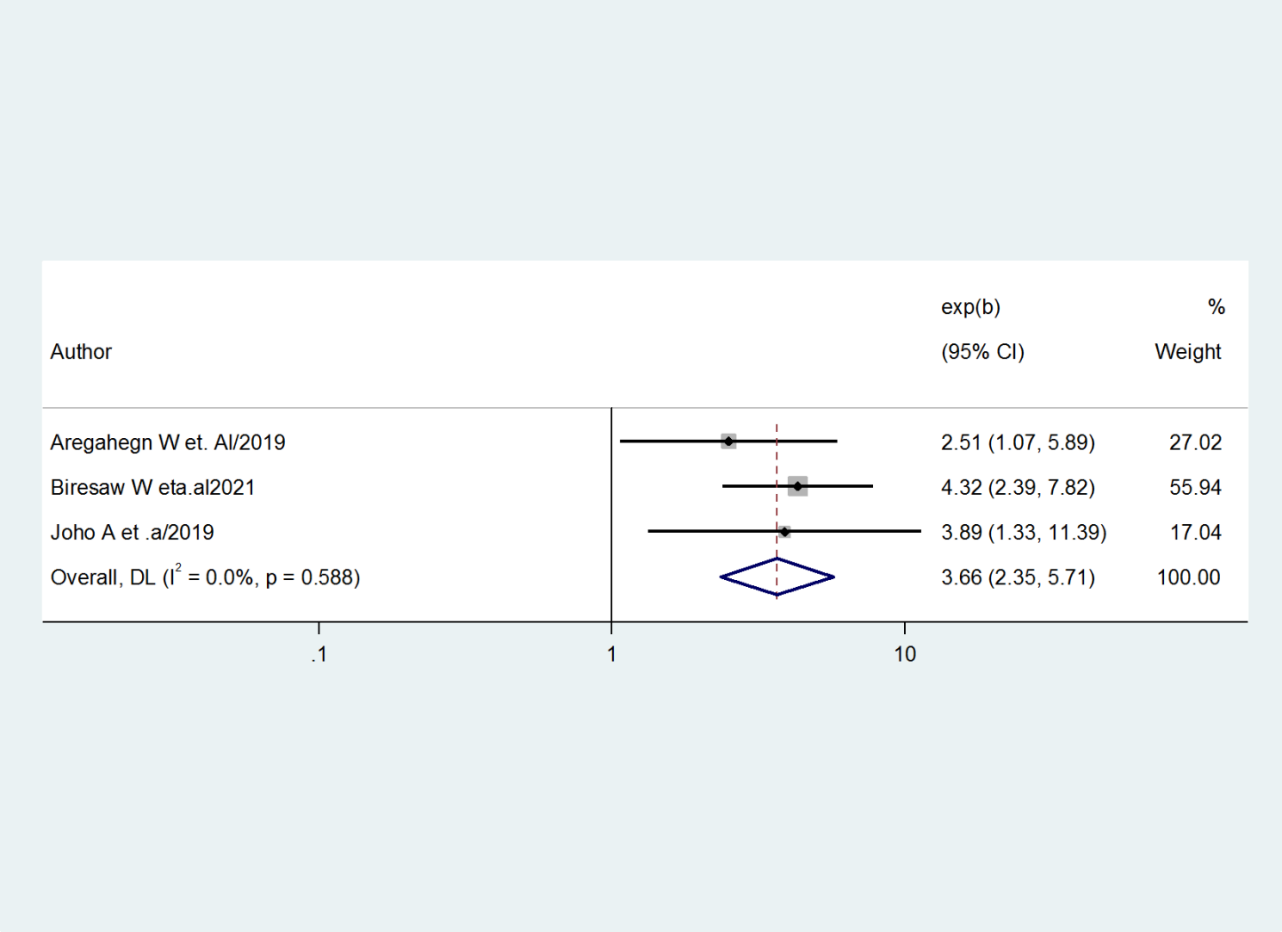
Forest plot showing the association between the practice of active management of the third stage of labor and years of experience

The association between the prevalence of the good practice of AMTSL and years of experience was evaluated by using three studies [22, 25, 27]. The results revealed that the pooled effect of years of experience was significantly associated with good practice for active management of the third stage of labor. Obstetric care providers who had six up to ten years of experience were almost four times more likely to practice for AMTSL than those having years of experience less than six years (AOR = 3.66, 95%CI = 2.35, 5.71). The studies did not show any evidence of heterogeneity (I-squared = 0.0%, p = 0.588) (Fig. 7).

## Discussion

The significance of each intervention in active management, which consists of several packaged therapies, is uncertain. Controlled cord traction which requires manual skill training to be done correctly. The third stage of labor would be handled quite differently at lower levels of healthcare if controlled cord traction could be discontinued without diminishing effectiveness. Since some AMTSL components require training while others call for an effective drug procurement and utilization system. The programmatic significance of the contribution of the various AMTSL components to the overall effect in reducing the incidence of hemorrhage may be significant.

The review was conducted to find any related gaps that would be generally very crucial to improving practice for active management of the third stage of labor in Sub-Saharan countries. This type of study will be important to managers of programs and leaders in countries that are developing like East Africa Countries to reduce maternal and neonatal mortality. More than 2,000 obstetric caregivers from three East African countries participated in our Systematic Review and Meta-analysis.

As a result, the pooled prevalence of practice for active management of the third stage of labor in East Africa was 34.42% (95% CI 25.48, 43.35). This is lower than the study conducted in (Kashmir) India (60.0%) [34], New Zealand (48.1%) [35], (Sikkim) India [36]. This finding is higher than the study conducted in Egypt (15%) [37]. However, it is consistent with a systematic review that was done in Ethiopia (39.65%) [38].

A subgroup study by region revealed that the practice prevalence in East Africa was 34.45%, and in West Africa was 61.67%. This indicated the pooled prevalence of knowledge for AMTSL in East African Countries is lower than in other Sub-Saharan Countries (Western and Southern African countries). The disparity may result from inequalities in maternity service delivery in Sub-Saharan African countries. Many research studies and organizations suggest that teamwork and preparation for childbirth are essential for managing labor efficiently and avoiding unexpected complications [39].

The factors that were associated with healthcare professionals’ practice of active management of the third stage of labor were received training, years of experience, and good knowledge. Obstetric care providers who had received in-service training were three times more likely to practice AMTSL (OR = 3.02, 95%CI = 2.13, 4.28). Obstetric care providers who had good knowledge were six times practice AMTSL than who had poor knowledge (OR = 6.25, 95%CI = 3.69, 10.58). Obstetric care providers who had six up to ten years of experience were almost four times more likely to practice for AMTSL than those having years of experience less than six years (OR = 3.66, 95%CI = 2.35, 5.71) [40].

Ministries of Health should implement policies and programs to ensure that every woman is provided a uterotonic immediately following birth, whether she gives birth in a facility with a trained provider or at home in the community. This is because there is clear evidence that the administration of a uterotonic is the most important aspect of AMTSL. This can be accomplished by promoting AMTSL in healthcare settings and creating community-based initiatives for the use of misoprostol for women who give birth at home. These kinds of initiatives can broaden coverage to ensure that almost all pregnant women remain safe from severe PPH [41].

In this review, no data are available on community-based misoprostol distribution for practicing AMTSL. However, the randomized control trial suggested that the need for task shifting of AMTSL may offer a chance for facility-based obstetric care to improve treatment quality due to the shortage of obstetric care providers, which is the final step of postpartum assessment of the uterine tonus can be effectively conducted by patients, whilst regularly monitored obstetric care providers [42]. Finally, it may be used as a source of information for researchers(conduct quasi-randomized control and qualitative studies: a limited number of studies conducted), program managers, policymakers, and other concerned bodies to design appropriate strategies that improve those obstetric care providers’ skills on AMTSL.

## Strengths and limitations of the study

To reduce selection bias, we conducted a systematic search of the literature and included research using well-defined criteria. Since we only examined English-language publications and some databases (including EMBASE and HINARI). Preprint articles, which are yet to undergo peer review, were also included. The results of these studies may therefore change in subsequent studies, and methodological biases may arise.

## Conclusion

This systematic review and meta-analyses revealed that the pooled prevalence of practice for AMTSL in East African countries was low. Moreover, the prevalence of practice differed by region. The following factors are statically associated with the practice of AMTSL: received training, years of experience, and good knowledge. Obstetric care providers should continue to receive training in all components of AMTSL through training and education programs. Furthermore, AMTSL should continue to be promoted through national policies, and systems should be in place to track and oversee its use.

## Electronic supplementary material

Below is the link to the electronic supplementary material.

**Supplementary 1**. PRISMA checklist

**Supplementary 2**. Quality assessment for the included Studies

**Supplementary 3**. Risk of bias assessment for the included studies

## Data Availability

The datasets used and/or analyzed during the current study are available from the corresponding author upon reasonable request.
