# Supplementary material for: Practices and factors associated with active management of the third stage of labor in East Africa: systematic review and meta-analysis
Source: BMC Pregnancy Childbirth. 2023 Jun 13;23:438. doi: 10.1186/s12884-023-05761-9 (PMC10265795; doi:10.1186/s12884-023-05761-9)
Supplement: Supplementary file 2 — Supplementary 2. Quality assessment for the included Studies [file 12884_2023_5761_MOESM2_ESM.docx]

Supplementary2. Quality assessment for the included Studies

| Item | Clearly defined inclusion | Describe study setting and participant | Valid and reliable exposure measurement | Objective and standard criteria for measurement | Identified confounder | Strategies to deal with confounders | Valid and reliable outcome measurement | Appropriate statically analysis | No of ‘yes’s ‘ |
| --- | --- | --- | --- | --- | --- | --- | --- | --- | --- |
| Rahel Y et.al | Yes | Yes | Yes | Yes | No | No | Yes | Yes | 6/8=75 |
| Biresaw W eta.al | Yes | Yes | No | Yes | Yes | Yes | Yes | Yes | 7/8=87.5 |
| Getu E et.al | Yes | Yes | Yes | Yes | Yes | No | Yes | Yes | 7/8=87.5 |
| Wondwosen M et. al | Yes | Yes | No | Yes | Yes | Yes | Yes | Yes | 7/8=87.5 |
| Aregahegn W et. al/ | Yes | Yes | No | Yes | Yes | No | Yes | Yes | 6/8=75 |
| Godfrey S et.al | Yes | Yes | No | Yes | Yes | No | Yes | Yes | 6/8=75 |
| Joho A et .al | Yes | Yes | Yes | Yes | Yes | Yes | Yes | Yes | 7/8=75 |
| Fatina B et.al | Yes | Yes | No | Yes | Yes | Yes | Yes | Yes | 7/8=85.5 |
| Muyanga D et.al | Yes | Yes | No | Yes | Yes | Yes | Yes | Yes | 7/8=85.5 |
| Fatina R et.al | Yes | Yes | No | Yes | Yes | Yes | Yes | Yes | 7/8=85.5 |
| Haule M/ | Yes | Yes | No | Yes | Yes | Yes | Yes | Yes | 7/8=85.5 |
| Sangay B et.al | Yes | Yes | No | Yes | Yes | Yes | Yes | Yes | 7/8=85.5 |
| Abalo J | Yes | Yes | No | Yes | Yes | Yes | Yes | Yes | 7/8=85.5 |
