# Supplementary material for: Practices and factors associated with active management of the third stage of labor in East Africa: systematic review and meta-analysis
Source: BMC Pregnancy Childbirth. 2023 Jun 13;23:438. doi: 10.1186/s12884-023-05761-9 (PMC10265795; doi:10.1186/s12884-023-05761-9)
Supplement: Supplementary file 3 — Supplementary 3. Risk of bias assessment for the included studies [file 12884_2023_5761_MOESM3_ESM.docx]

**Supplementary3**. Risk of bias assessment for the included studies

| Item | External validity | | | | Internal validity | | | | | |  | |
| --- | --- | --- | --- | --- | --- | --- | --- | --- | --- | --- | --- | --- |
|  | Representativenes s of the target population | Representativenes s of the sampling frame | Radom samplin g or census | Minimal respons e bias | Data were collecte d directly | Acceptabl e case definition used in the study | Valid and reliable measurement t | The same mode of data collectio n for all study subject | Appropriat e length of prevalence period for parameter of interest | Appropriate numerators and denominator s of interest | No of yes | **Summ ary of risk of bias** |
| Rahel Y et.al/ | Yes | Yes | Yes | Yes | No | No | Yes | Yes | Yes | Yes | 8 | Loiw risk |
| Biresaw W eta.al | Yes | Yes | No | Yes | Yes | No | Yes | Yes | Yes | Yes | 8 | Low- risk |
| Getu E et.al | Yes | Yes | No | Yes | Yes | Yes | Yes | Yes | Yes | Yes | 9 | Low – risk |
| Wondwosen M et. Al | Yes | Yes | No | Yes | Yes | Yes | Yes | Yes | Yes | Yes | 9 | Low- risk |
| Aregahegn W et. A | Yes | Yes | Yes | Yes | Yes | No | Yes | Yes | Yes | Yes | 9 | Low- risk |

| Godfrey S et.al | Yes | Yes | Yes | Yes | Yes | Yes | Yes | No | Yes | Yes | 9 | Low-  risk |
| --- | --- | --- | --- | --- | --- | --- | --- | --- | --- | --- | --- | --- |
| Joho A et .al | Yes | Yes | Yes | Yes | Yes | Yes | No | Yes | Yes | Yes | 8 | Low- risk |
| Fatina B et.al | Yes | Yes | No | Yes | Yes | Yes | Yes | Yes | Yes | Yes | 9 | Low risk |
| Muyanga D et.al | Yes | Yes | Yes | Yes | Yes | Yes | Yes | Yes | No | No | 8 | Low- risk |
| Fatina R et.al | Yes | Yes | Yes | Yes | Yes | Yes | Yes | No | No | Yes | 8 | Low- risk |
| Haule M | Yes | Yes | Yes | Yes | Yes | Yes | Yes | Yes | Yes | No | 9 | Low- risk |
| Sangay B et.al | Yes | Yes | Yes | No | Yes | Yes | Yes | No | Yes | Yes | 8 | Low -risk |
| Abalo J |  | Yes | Yes | No | Yes | Yes | Yes | No | Yes | Yes | 8 | Low risk |
